# Establishment of an *Agrobacterium*‐mediated CRISPR/Cas9 Genome Editing System for Kenaf (*Hibiscus cannabinus*)

**DOI:** 10.1111/pbi.70657

**Published:** 2026-04-03

**Authors:** Xueqing Pan, Lingling Zhuang, Siyan Wu, Chuanyu Wang, Qin Li, Jianmin Qi, Pingping Fang, Jiantang Xu, Aifen Tao, Shuangxia Jin, Liwu Zhang

**Affiliations:** ^1^ Key Laboratory of Ministry of Education for Genetics, Breeding and Multiple Utilisation of Crops Fujian Agriculture and Forestry University Fuzhou China; ^2^ National Key Lab of Crop Genetic Improvement Huazhong Agricultural University Wuhan China

**Keywords:** *agrobacterium*‐mediated transformation, genome editing, hairy root system, kenaf

Kenaf ( *Hibiscus cannabinus* L.), an annual herbaceous species within the Hibiscus genus in the Malvaceae family, is a globally significant fibre crop and a pivotal industrial resource for producing rope, paper, building materials, absorbents and so on (Zhang et al. 2020). While the CRISPR/Cas9 system has been extensively utilised for genetic improvement in major crops such as rice (Shi et al. 2025), wheat (Debernardi et al. 2020) and cotton (Xu et al. 2025), its application in kenaf remains largely unexplored. This limitation significantly hinders the utility of CRISPR/Cas9 for functional genomic studies in kenaf.

The kenaf cultivar ‘Fuhong 952’ with the reference genome was used in this study. *Agrobacterium rhizogenes* K599 (unloaded) was employed to infect shoot tips, cotyledon petioles and hypocotyls excised from 7 to 10‐day‐old sterile seedlings (Figure S1). After 15 days, rooting parameters—including roots number, roots length and rooting rate—were assessed for each explant type (Figure S2). Cotyledon petioles and shoot tips exhibited the highest rooting rates (nearly 100%); cotyledon petioles produced the longest roots and the greatest number of root shoots. A composite root vigour index confirmed cotyledon petioles as the optimal explant for establishing a hairy root induction system in kenaf.

The transformation efficiency of K599 was further evaluated using the reporter genes pCMV::GUS and pCMV::DsRed2 (Figure 1a). GUS histochemical staining revealed an extremely high transformation efficiency of regenerated hairy roots, approaching 100% per plant (Figure S3). To systematically investigate the effects of various infection parameters on *A. rhizogenes* transformation efficiency, each parameter was subjected to gradient adjustments in a factorial experimental design. GUS histochemical staining demonstrated that the optimal transformation efficiency was achieved under the following conditions: a bacterial suspension concentration of OD_600 _= 1.0, a 20 min infection duration and a co‐cultivation temperature of 22°C (Figure S4). These conditions were subsequently adopted as the standard protocol for functional validation experiments.

**FIGURE 1:**
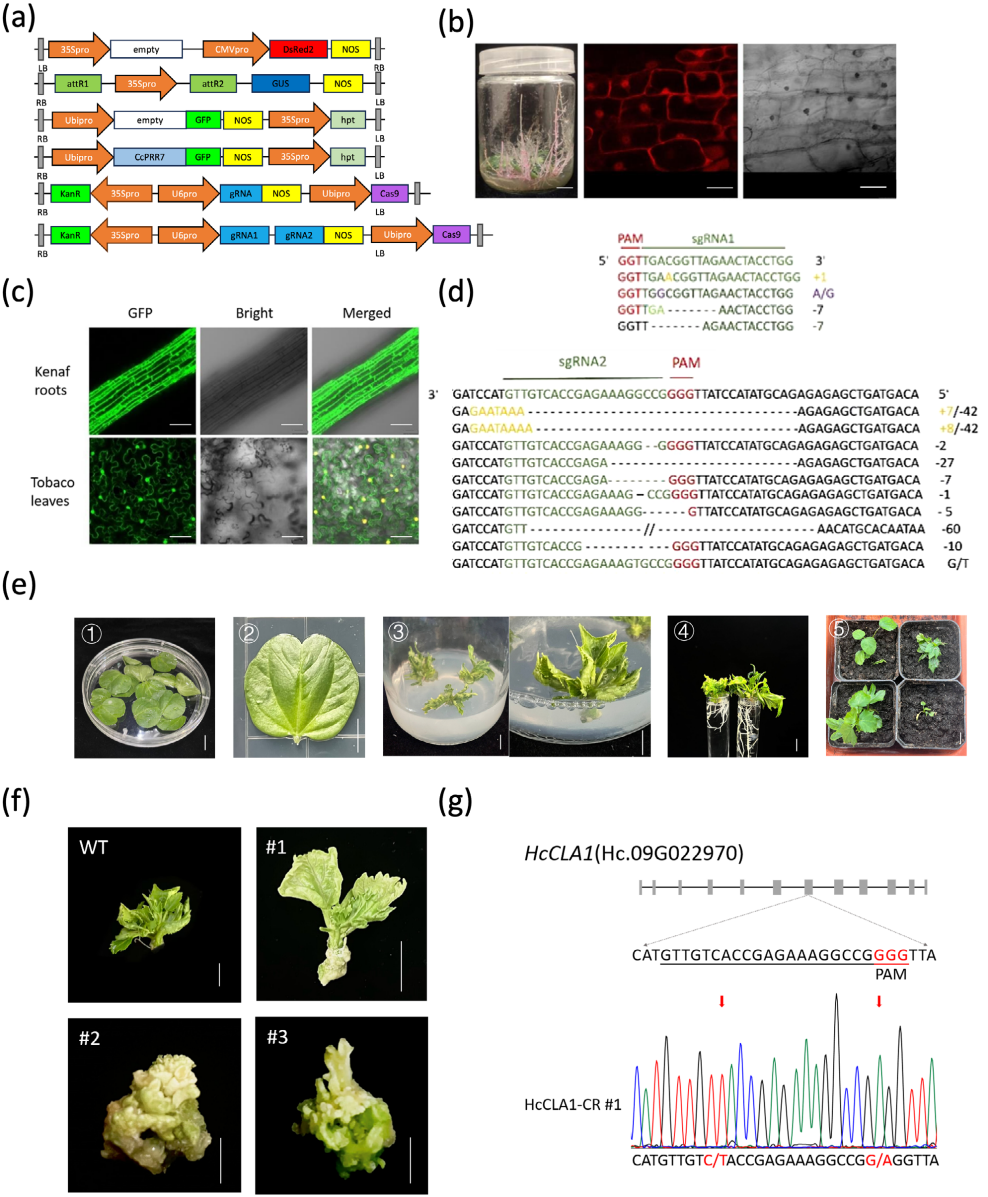
Establishment of a hairy root induction system (a–d) and CRISPR/Cas9‐mediated genome editing of the *HcCLA1* gene (e to g) in kenaf. (a) Schematic representation of the vector constructs used in this study. (b) Growth of hairy roots induced by *Agrobacterium rhizogenes* strain K599. Scale bar, 1 cm. Transgenic‐positive roots exhibiting red fluorescence are shown at the middle. Scale bar, 1 cm. (c) Subcellular localisation of CcPRR7‐GFP fusion protein in kenaf roots and tobacco leaves. Scale bar, 25 μm. (d) Mutations induced by *pGhU6.7*::*gRNA1* and *pGhU6.7*::*gRNA2* at the *HcCLA1* target site. The PAM sequence is highlighted in red and the target site is shown in green. (e) Protocol for stable genetic transformation of kenaf mediated by *Agrobacterium tumefaciens strain* GV3101: 1 Explant infection for 20 min. 2 Cotyledon petiole induction for 40–45 days. 3 Regeneration of seedlings for 10–15 days. 4 One‐day acclimation at room temperature. 5 Transfer of regenerated seedlings to soil. (f) Phenotypic characterisation of albino seedlings regenerated from callus tissues. Scale bar, 1 cm. (g) Sanger sequencing chromatograms revealing CRISPR/Cas9‐mediated editing at the gRNA2 target site in *HcCLA1* from albino seedling #1. The PAM site is indicated by a red box.

Transgenic roots were validated using the DsRed2 fluorescent reporter (Figure 1b). In *CcPRR7* (Cc.03G0003190), overexpressing kenaf hairy roots, bright nuclear and membrane‐localised fluorescence was observed, consistent with its tobacco‐based localisation (Figure 1c). Notably, the stable hairy root system yielded sharper signals than transient assays. *HcMYB113* (Hc.11G004950), selected for its conserved role in flavonoid/anthocyanin biosynthesis and co‐expression with *HcTT8a* in secondary metabolism‐related transcriptome data, was confirmed to interact with HcTT8a (Hc.08G021280) in vivo via bimolecular fluorescence complementation (BiFC) (Figure S5). Collectively, the robust K599‐mediated hairy root system established in kenaf enabled efficient validation of subcellular localisation, protein–protein interactions and molecular regulatory mechanisms.

CRISPR/Cas9‐mediated genome editing enables precise gene modification for plant functional genomics. The GhU6.7 and GhU6.9 snRNA promoters, isolated from cotton ( *Gossypium hirsutum* ) (Wang et al. 2018)—a Malvaceae species closely related to kenaf—were selected due to potential functional conservation. Sequence analysis confirmed conserved upstream sequence elements (USEs) and TATA‐box motifs characteristic of plant U6 promoters (Figure S6). These promoters were used to construct a multi‐target CRISPR/Cas9 system targeting the *HcCLA1* gene (Hc.09G022970) with two gRNAs: gRNA1 (5′‐GGTCCATCAAGATTGGCAGT‐3′) and gRNA2 (5′‐GTTGTCACCGAGAAAGGCCG‐3′) (Figure 1a). The resulting vectors, *pGhU6.7::gRNA‐Ubi::Cas9* and *pGhU6.9::gRNA‐Ubi::Cas9*, were introduced into kenaf cotyledon petioles via *Agrobacterium rhizogenes* K599 infection. The GhU6.7‐driven construct successfully generated 52 independent transgenic hairy root lines.

Genomic DNA from K599‐infected hairy roots was analysed using Cas9‐specific primers, confirming T‐DNA integration in 34 of 52 independent transgenic lines (Figure S7). TA‐cloning and Sanger sequencing of amplicons spanning both sgRNA target sites revealed mutations at both loci in 14 lines, corresponding to an overall editing efficiency of 41.2% (14/34) (Table S1). Mutations predominantly comprised 1–5 bp insertions, substitutions and deletions at the predicted cleavage sites (Figure 1d). Phenotypically, *HcCLA1*‐knockout roots exhibited significantly reduced length and root number relative to empty‐vector controls (Figure S8), confirming functional disruption. In contrast, parallel experiments using the GhU6.9 promoter yielded substantially lower efficiency at 9.7% (3/31) (Table S1). The 4.2‐fold higher efficiency conferred by GhU6.7 underscores promoter selection—likely influencing transcriptional activity or sgRNA processing—as a critical determinant of CRISPR efficacy in kenaf. These findings establish GhU6.7 as a preferred promoter for CRISPR‐based genome engineering in this species.

*HcCLA1*, a key regulator of chloroplast development, serves as a visible marker for transformation given its disruption‐induced albino phenotype. Leveraging this trait, we developed a stable *Agrobacterium tumefaciens* (GV3101)‐mediated transformation system for kenaf ( *Hibiscus cannabinus* ) using cotyledon petioles as explants. The binary vector *pGhU6.7::gRNA(HcCLA1)‐Ubi::Cas9* was introduced via an optimised protocol comprising five steps: (1) 20‐min infection, (2) 48‐h co‐cultivation, (3) callus and shoot induction (40–45 days), (4) shoot elongation (10–15 days) and (5) root induction (10–15 days) (Figure 1e, Table S2). Of 751 regenerated plantlets, three albino mutants were confirmed by Cas9‐specific PCR (Figure 1f, Table S3). Sequencing of a 1000 bp fragment spanning both *HcCLA1* target sites revealed consistent single‐nucleotide mutations at the gRNA2 target site in all three albino lines, whereas the controls showed no sequence alterations (Figure 1g).

The low stable transformation efficiency in kenaf primarily from a poor bud induction rate in explants. To address this, we propose integrating bud regeneration‐inducing factors into the induction medium and systematic optimisation of hormone ratios by testing multiple concentration gradients per component, employing orthogonal experiments or response surface methodology (RSM) to determine the optimal balance for shoot regeneration. This strategy has proven successful in recalcitrant crops such as soybean and cotton and is anticipated to yield similar improvements in kenaf. Notably, our integrated approach—combining a rapid hairy root system with stable transformation and achieving over 40% editing efficiency via promoter optimisation—constitutes a significant technical advancement for kenaf, a fibre crop historically underexplored in genetic research.

The CRISPR/Cas9 platform developed herein holds the promise for kenaf genetic improvement, particularly for enhancing fibre quality via targeted modification of secondary cell wall biosynthesis genes and developing salt‐tolerant varieties through knock‐in of beneficial alleles. While the GhU6.7 promoter achieved the highest editing efficiency in hairy roots, the current rate remains suboptimal for routine stable transformation. Future work will focus on utilising tissue‐specific promoters to spatially and temporally regulate Cas9 expression, thereby minimising off‐target effects and improving transformation viability. Additionally, codon‐optimised Cas9 variants and advanced delivery methods will be explored to further refine the genome‐editing platform in kenaf.

## Funding

This work was supported by the National Natural Science Foundation of China, 32472219. Fujian Natural Science Foundation of China, 2023J01443. China Agriculture Research System of the Ministry of Agriculture and MARA, CARS‐16. Science and Technology Innovation Project of Fujian Agriculture and Forestry University, KFB23001, KFB24080.

## Supporting information


**Figure S1:** Rooting efficiency at different stages after infection of different explants in kenaf with unloaded K599.
**Figure S2:** Statistical analysis of regeneration root indices induced by different explant types.
**Figure S3:** β‐Glucuronidase (GUS) histochemical staining of transgenic hairy roots.
**Figure S4:** Effects of infection duration, *Agrobacterium* suspension concentration and co‐cultivation temperature on GUS staining efficiency in K599‐infected cotyledon petioles.
**Figure S5:** Bimolecular fluorescence complementation (BiFC) assays in kenaf hairy roots.
**Figure S6:** Comparison of homology between GhU6.7 and GhU6.9 promoters.
**Figure S7:** Detection of Cas9 gene integration in hairy roots using a specific primer pair.
**Figure S8:** Phenotypic comparison between *HcCLA1*‐transgenic hairy roots (four right ones) and empty‐vector‐transformed control roots (the left one).

**Table S1:** Editing efficiencies in kenaf hairy roots mediated by two U6 promoters.
**Table S2:** Composition of culture media used for hairy root induction and stable transformation of kenaf.
**Table S3:** Primers used in this study.

**Data S1:** pbi70657‐sup‐0003‐Supinfo.docx.

## Data Availability

The data that supports the findings of this study are available in the Supporting Information of this article.
