# Supplementary material for: Establishment of an Agrobacterium‐mediated CRISPR/Cas9 Genome Editing System for Kenaf (Hibiscus cannabinus)
Source: Plant Biotechnol J. 2026 Apr 3;24(7):4719–21. doi: 10.1111/pbi.70657 (PMC13278532; doi:10.1111/pbi.70657)
Supplement: Supplementary file 1 — Figure S1: Rooting efficiency at different stages after infection of different explants in kenaf with unloaded K599. Figure S2: Statistical analysis of regeneration root indices induced by different explant types. Figure S3: β‐Glucuronidase (GUS) histochemical staining of transgenic hairy roots. Figure S4: Effects of infection duration, Agrobacterium suspension concentration and co‐cultivation temperature on GUS staining efficiency in K599‐infected cotyledon petioles. Figure S5: Bimolecular fluorescence complementation (BiFC) assays in kenaf hairy roots. Figure S6: Comparison of homology between GhU6.7 and GhU6.9 promoters. Figure S7: Detection of Cas9 gene integration in hairy roots using a specific primer pair. Figure S8: Phenotypic comparison between HcCLA1‐transgenic hairy roots (four right ones) and empty‐vector‐transformed control roots (the left one). [file PBI-24-4719-s003.docx]

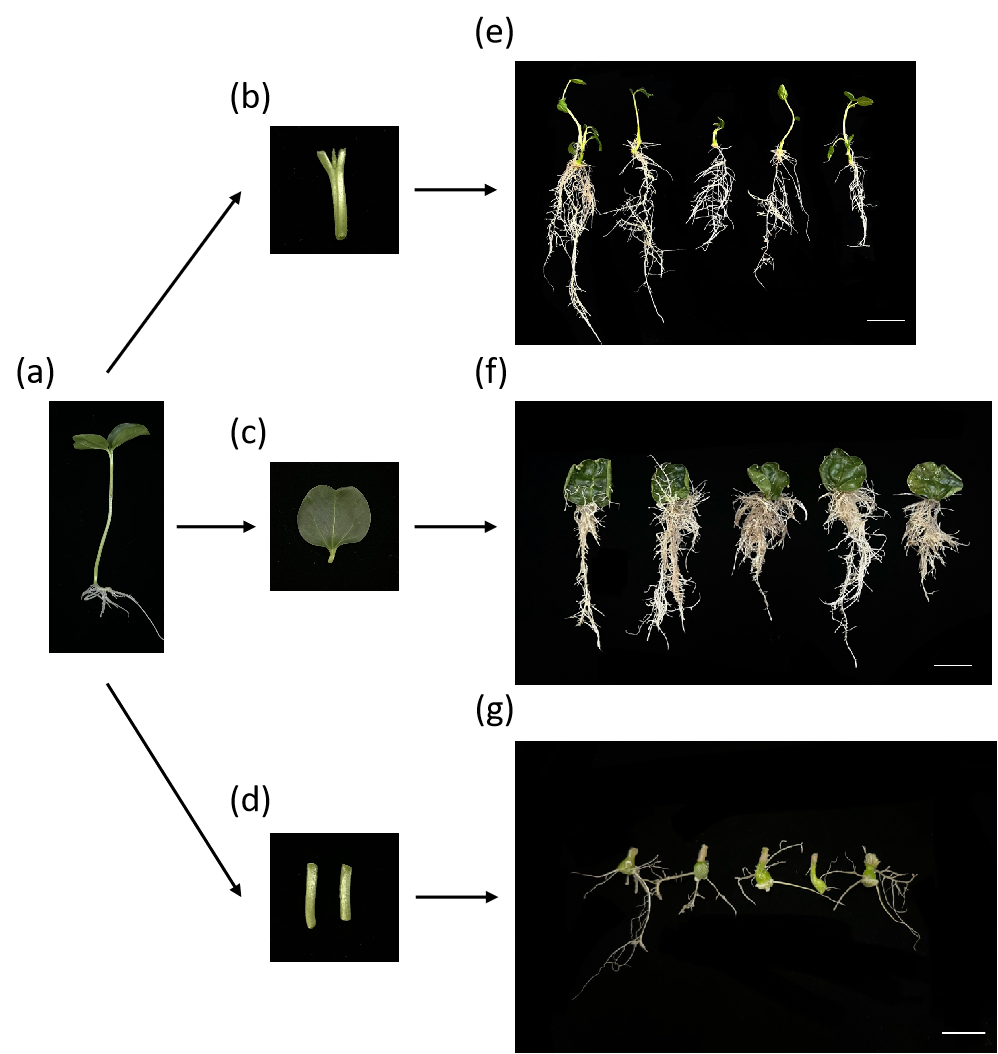


**Figure S1** **Rooting efficiency at different stages after infection of different explants in kenaf with unloaded K599.**

(a) The 5-day-old sterile kenaf seedling used as the source for taking different explants.

(b-d) Three types of explants (b-shoot tips, c-cotyledon petioles, d-hypocotyls) excised from the 7-10-day-old sterile kenaf seedling shown in (a).

(e-g) Hairy roots derived from three types of explants (e-shoot tips, f-cotyledon petioles, g-hypocotyls) infected by *Agrobacterium rhizogenes* K599 after 15 days of induction. These images demonstrate the successful induction of hairy roots from each explant type, highlighting the differences in rooting efficiency among them.


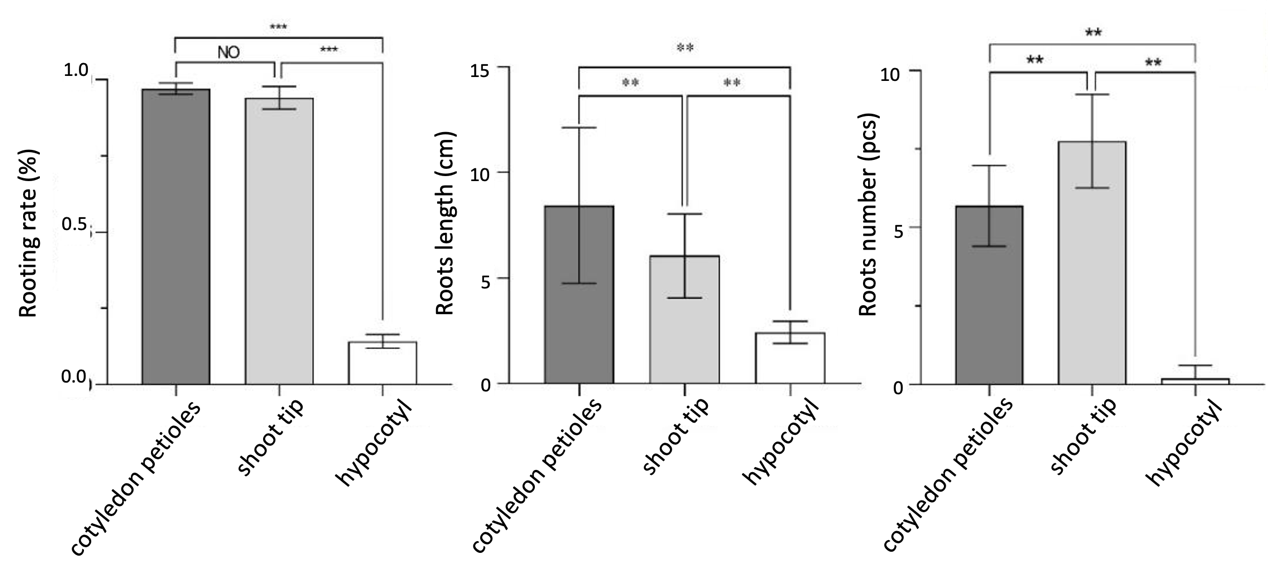


**Figure S2** **Statistical analysis of regeneration root indices induced by different explant types.**

All data points represent mean ± standard error (n ≥ 3). **p < 0.01, ***p < 0.001

**
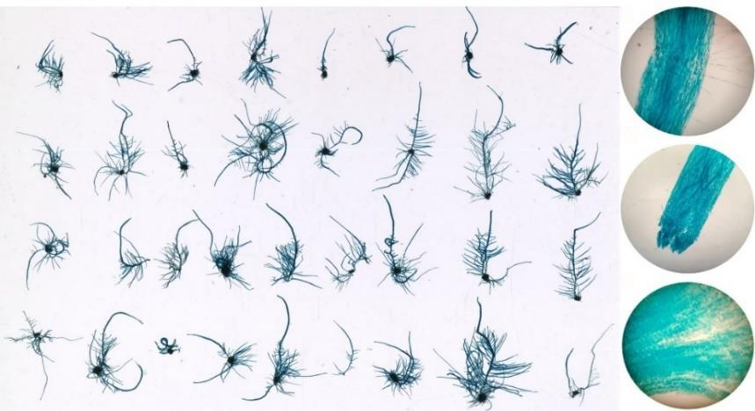
**

**Fig S3** **β‐Glucuronidase (GUS) histochemical staining of transgenic hairy roots.**


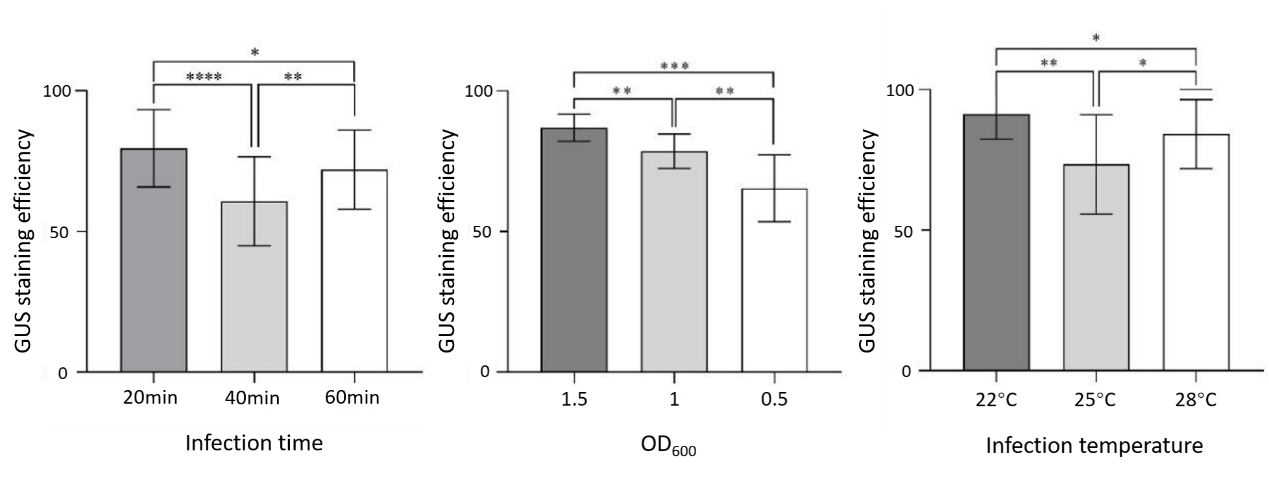


**Figure S4** **Effects of infection duration, *Agrobacterium* suspension concentration and co‐cultivation temperature on GUS staining efficiency in K599‐infected cotyledon** **petioles.**

All data points represent mean ± standard error (n ≥ 3). *p < 0.05, **p < 0.01, ***p < 0.001.


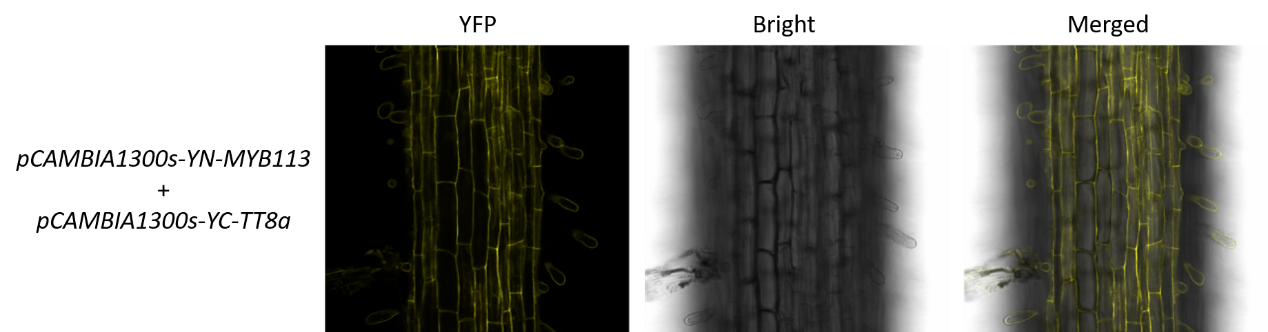


**Fig S5** **Bimolecular fluorescence complementation (BiFC) assays in kenaf hairy roots.**

**
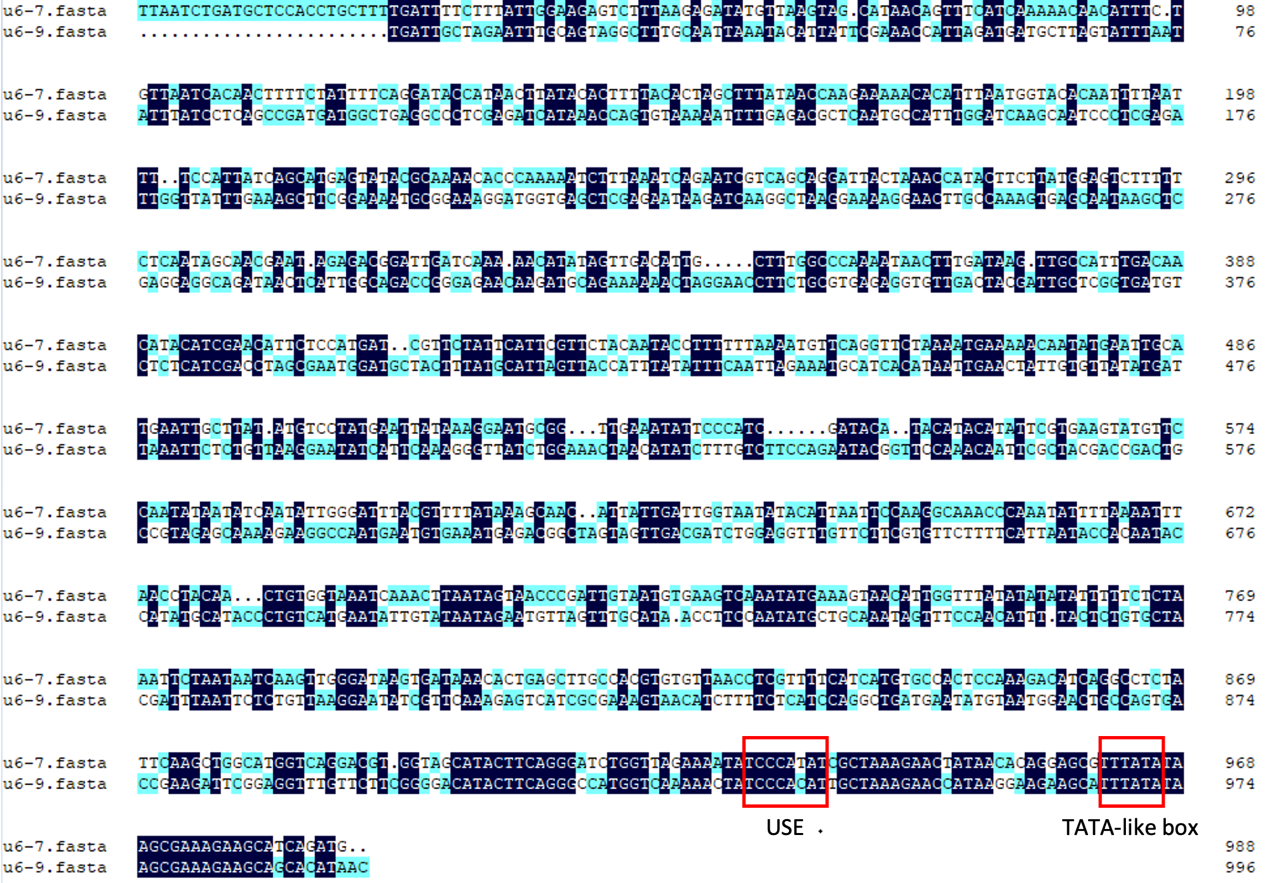
**

**Fig S6 Comparison of homology between GhU6.7 and GhU6.9 promoters.**

**
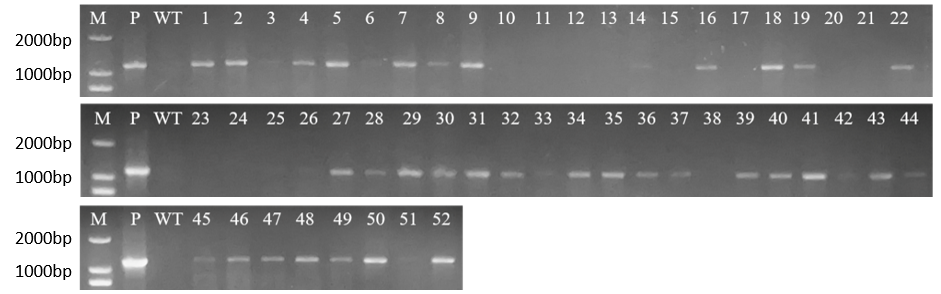
**

**Fig S7** **Detection of Cas9 gene integration in hairy roots using a specific primer pair.** Expected fragment size: 1,144 base pairs (bp).

**
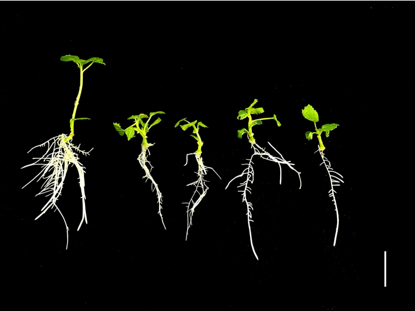
**

**Fig S8** **Phenotypic comparison between *HcCLA1* - transgenic hairy roots (four right ones) and empty-vector-transformed control roots (the left one).**
