# Supplementary material for: Establishment of an Agrobacterium‐mediated CRISPR/Cas9 Genome Editing System for Kenaf (Hibiscus cannabinus)
Source: Plant Biotechnol J. 2026 Apr 3;24(7):4719–21. doi: 10.1111/pbi.70657 (PMC13278532; doi:10.1111/pbi.70657)
Supplement: Supplementary file 2 — Table S1: Editing efficiencies in kenaf hairy roots mediated by two U6 promoters. Table S2: Composition of culture media used for hairy root induction and stable transformation of kenaf. Table S3: Primers used in this study. [file PBI-24-4719-s002.docx]

Table S1 Editing efficiencies in kenaf hairy roots with two U6 promoters.

| U6 promoter | Number of explants | Number of explants successfully induced by hairy roots | Number of explants with *Cas9* gene detected in hairy roots | Number of explants with editing events in hairy roots | Genome editing efficiency of hairy roots |
| --- | --- | --- | --- | --- | --- |
| *GhU6.7* | 56 | 52 | 34 | 4 (gRNA1) | 1.2% |
|  |  |  |  | 10 (gRNA2) | 29.4% |
| *GhU6.9* | 50 | 38 | 31 | 0 (gRNA1) | 0 |
|  |  |  |  | 3 (gRNA2) | 9.6% |

Table S2 Composition of culture media used for hairy root induction and stable transformation of kenaf.

| **Medium** | **Abbreviation** | **Composition (per liter)** |
| --- | --- | --- |
| Murashige and Skoog (MS) Seed Germination Medium | SGM | MS Basal Salts and Vitamins (4.4 g), sucrose (30.0 g), Agar (8.0 g), pH = 5.8 |
| MS infection Medium | INM | MS Basal Salts and Vitamins (4.4 g), sucrose (30 g), Acetosyringone (100 µM), pH = 5.6-5.7 |
| MS Hairy Root Co-Cultivation Medium | HRCM | MS Basal Salts and Vitamins (4.4 g), sucrose (30 g), Acetosyringone (100 µM), pH = 5.8 |
| MS Hairy Root Antibacterial Medium | HRAM | MS Basal Salts and Vitamins (4.4 g), sucrose (30.0 g), Agar (8.0 g), Timentin (100 mg), pH = 5.8 |
| MS Hairy Root Induction Media | HRIM | MS Basal Salts and Vitamins (4.4 g), sucrose (30.0 g), Agar (8.0 g), Timentin (100 mg), pH = 5.8 |
| MS Callus and Shoots Induction Medium | CSIM | MS Basal Salts and Vitamins (4.4 g), sucrose (30.0 g), Agar (8.0 g), TDZ (0.2-0.4 mg), 2,4-D (0.1-0.2 mg), Timentin (100 mg), pH = 5.8 |
| MS Selective Pressure Medium | SPM | MS Basal Salts and Vitamins (4.4 g), sucrose (30.0 g), Agar (8.0 g), TDZ (0.2-0.4 mg), 2,4-D (0.1-0.2 mg), Kanamycin (50 mg), Timentin (100 mg), pH = 5.8 |
| MS Shoot Elongation Medium | SEM | MS Basal Salts and Vitamins (4.4 g), sucrose (30.0 g),Agar(8.0 g), TDZ (0.2-0.4 mg), 2,4-D (0.1-0.2 mg), Timentin (100 mg), pH = 5.8 |
| MS Rooting Medium | ROM | MS Basal Salts and Vitamins (4.4 g), sucrose (30.0 g), Agar (8.0 g), Timentin (100 mg), pH = 5.8 |

MS Basal Salts and Vitamins: Murashige & Skoog Basal Medium with Vitamins (Lot: HCE0519387A), PhytoTechnology Laboratories, Lenexa, KS, USA.

Table S3 Primers used in this study.

| **Primer name** | | **Sequence (5**'–**3**'**)** | | **Usage** |
| --- | --- | --- | --- | --- |
| P-GFP-F  P-GFP-R | TTCTCGTTGGGGTCTTTGC  GACGACGGCAACTACAAGA | | *GFP* gene | |
| P-Red-F  P-Red-R | ATGTAAGGGCGGAAAGTAACC  GGGAAGGACAGCTTCTTGTAGTC | | *Red* gene | |
| P-PRR7-OE-F  P-PRR7-OE-R | TGCTGCTTCATGTGGTCGGG  TCAGGCCAGAGCGGTTTTTG | | Overexpression vector of *CcPRR7* | |
| P-Cas9-F  P-Cas9-R | GAGAATGAAGCGGATCGAA  CACACTCTTCAGTTTCTTGG | | *Cas9* gene | |
| P-CLA1-F  P-CLA1-R | CCGGGTTGGTTTGTATGGCT  GACTGGGTAACAGAACTGCCT | | Target site of the *HcCLA1* gene | |
