# Supplementary material for: Establishment of an Agrobacterium‐mediated CRISPR/Cas9 Genome Editing System for Kenaf (Hibiscus cannabinus)
Source: Plant Biotechnol J. 2026 Apr 3;24(7):4719–21. doi: 10.1111/pbi.70657 (PMC13278532; doi:10.1111/pbi.70657)
Supplement: Supplementary file 3 — Data S1: pbi70657‐sup‐0003‐Supinfo.docx. [file PBI-24-4719-s001.docx]

**Acknowledgments**

This work was supported by the National Natural Science Foundation of China (32472219), the Fujian Natural Science Foundation of China (2023J01443), the China Agriculture Research System of the Minishotry of Agriculture and MARA (CARS-16), and the Science and Technology Innovation Project of Fujian Agriculture and Foresty University (KFB23001, KFB24080).

This work was also supported by the platforms of Key Laboratory of Ministry of Agriculture and Rural Affairs for Biological Breeding of Fujian and Taiwan Crops, Fujian Key Laboratory for Crop Breeding by Design, Experiment Station of Ministry of Agriculture and Rural Affairs for Jute and Kenaf Scientific Observation in Southeast China, Public Platform of Fujian for Germplasm Resources of Bast Fiber Crops, Fujian International Science and Technology Cooperation Base for Genetic Breeding and Multiple Utilization Development of Southern Economic Crops.

**Author contributions**

**Xueqing Pan:** Investigation, Visualization, Writing - Original Draft, Writing - Review & Editing. **Lingling Zhuang:** Methodology, Investigation, Data Curation, Formal analysis. **Chuanyu Wang, Siyan Wu:** Methodology, Investigation. **Jianmin Qi, Pingping Fang, Jiantang Xu, Aifen Tao:** Resources. **Qin Li:** Methodology, Investigation, Writing - Review & Editing. **Shuangxia Jin:** Methodology, Supervision. **Liwu Zhang:** Methodology, Supervision, Funding acquisition, Writing - Review & Editing.
